# Urban mental health of children and youth: how cities affect our brains

**DOI:** 10.1192/j.eurpsy.2025.1182

**Published:** 2025-08-26

**Authors:** J. Vecerina, Z. Kušević

**Affiliations:** 1 Diagnosis and treatment, Zagreb child and youth protection center; 2 Clinic for child and adolescent psychiatry, University hospital center Zagreb, Zagreb, Croatia

## Abstract

**Introduction:**

This literature review will present the results obtained by searching available databases on the connection of everyday life in urban environments with the occurrence of symptoms of anxiety and depression, the use of addictive substances, schizophrenia as well as the occurrence of suicide in children, adolescents, and young adults. The results will be compared to the occurrence in suburban and rural areas.

**Objectives:**

The work aimed to clarify the hitherto known risk and protective factors associated with everyday life in the urban environment and to show how its adaptation has the potential to promote and protect the mental health of children and adolescents.

**Methods:**

Search of available databases: Medline, Scopus, PubMed.

**Keywords:**

Urban mental health; Adolescent mental health; Environment and health; Urban design for mental health;

**Results:**

The results of the presented studies indicate an ambivalent influence of the urban environment on the mental health of young people: on the one hand, better education and employment opportunities, easier choice of social circles, accessibility to health care services (especially if all of the mentioned services are accessible to young people independently through active mobility) contributes to the improved feeling of mental well-being. On the other hand, fewer green spaces, oversaturation with sound and visual stimuli, as well as involuntary interactions with other citizens can endanger mental health.

The collected data indicates a greater number of young people and adults with depression, anxiety symptoms, and psychotic symptoms in cities, while the frequency of attempted and committed suicides is more common in rural areas.

Many different interventions in the urban environment can improve the day-to-day experience and cumulative effect of city streets on our mental health. Such interventions in the built environment include increasing the amount of space dedicated to young people (especially young women) at the same time with the placement of green areas, decreasing automobile dependency, and improving active mobility infrastructure and public transport safety and efficiency.

**Image:**

**Figure:**
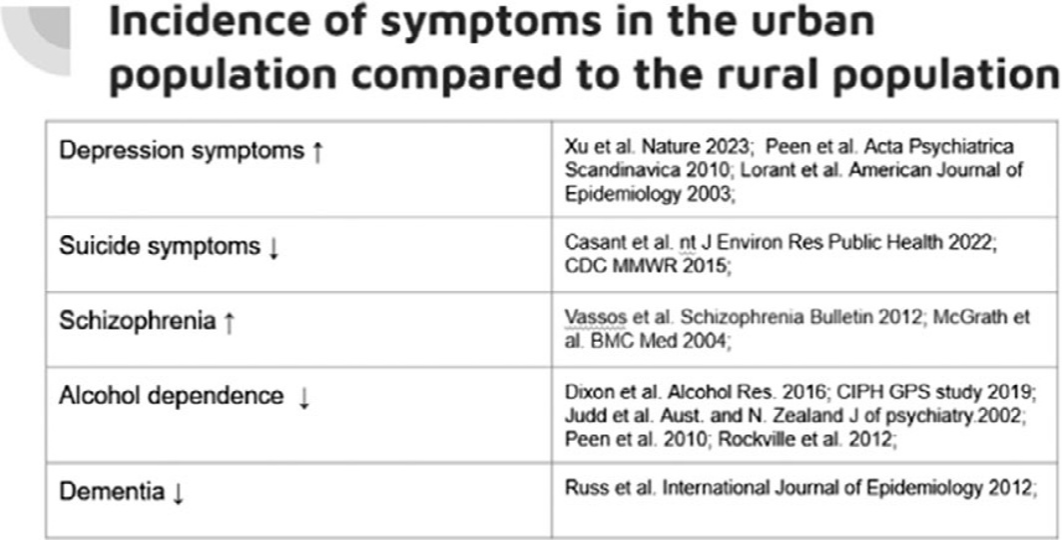

**Image 2:**

**Figure:**
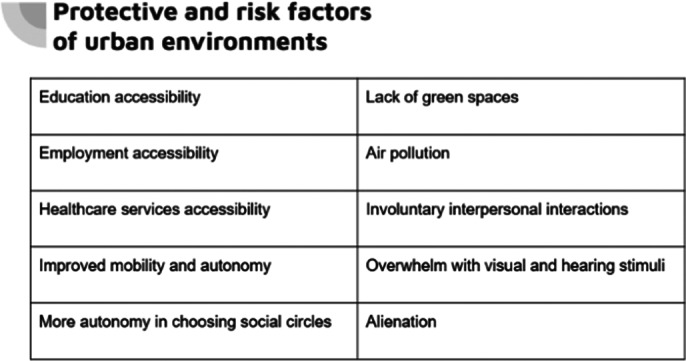

**Image 3:**

**Figure:**
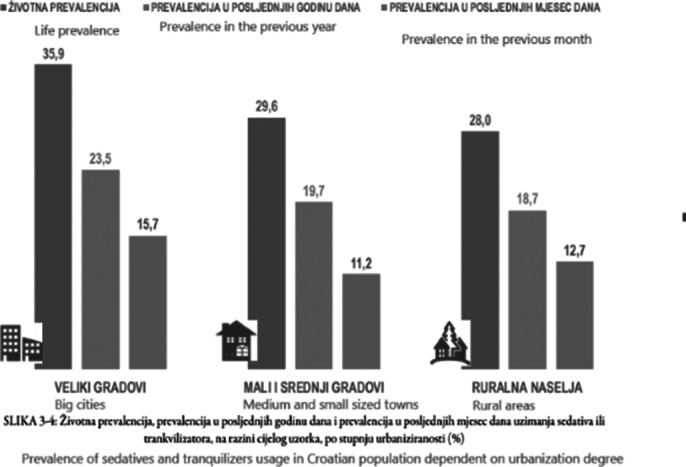

**Conclusions:**

Urbanization is related to the mental health of citizens, especially young people, and it is important to follow the guidelines when designing the public space of urban areas, which relate to the presence of green spaces, opportunities for active movement, and the existence of places that enable and encourage socialization, all with the perception security.

The results known so far should be kept in mind during further research into the etiology and epidemiology of mental health in young people, starting with epigenetics and ending with healthcare planning.

**Disclosure of Interest:**

None Declared

